# Supplementary material for: Association of menopausal status with body composition and anthropometric indices in Korean women
Source: PLoS One. 2024 May 20;19(5):e0298212. doi: 10.1371/journal.pone.0298212 (PMC11104629; doi:10.1371/journal.pone.0298212)
Supplement: Attachment — Submitted filename: Response to Reviewers-final.docx [file pone.0298212.s001.docx]

**Response to Reviewers**

To the Reviewers and Editor:

We appreciate the valuable comments that we received regarding our manuscript titled “Association of menopausal status with body composition and anthropometric indices in Korean women”. The manuscript was again revised by an English language editing service (American Journal Experts) to improve the clarity and readability of the English language in the paper. We have revised our manuscript in accordance with the Editors’ and Reviewers’ comments. Our revisions are listed below.

**Additional Editor Comments**:

Dear authors

Kindly do the corrections suggested by the reviewers and resubmit it.

**Reviewer #1:**

1. can the author please indicate the objective of the study clearly

**Answer**: As suggested by the reviewer, we have modified the objective of this study in Introduction section. The added contents are as follows:

“These conflicting results on the association between menopausal status and obesity may result in confusion. Therefore, the objective of the present cross-sectional study was to examine the association of menopausal status with anthropometric and body composition indices in South Korean women and to determine whether menopause status affects obesity or adiposity.”

2. what is the clinical implication/ impact of this study on clinical application.

**Answer**: We agreed with reviewer’s comment. Therefore, we have added the clinical implication of our study. The added contents are as follows:

“In this study, the clinical implication is that menopausal status is not associated with an increase of obesity or fat mass in any particular region of the body, but it is associated with skeletal muscle mass and total body water. Although many anthropometric and body composition indices were related to menopausal status, these associations disappeared after adjustment for age. Also, menopausal status induces the decrease of skeletal muscle mass and total body water, irrespective of age [28, 29]. But, until now, association of menopausal status with obesity or adiposity is still debatable. We think that the cause of the debate is due to aging [20, 23], physical activity [14, 25], and/or hormone changes [13, 26] in midlife women.”

3. the results can be better presented by standardizing the decimal point

**Answer**: We agree with reviewer’s comment. Therefore, we have modified the decimal point for standardization throughout all tables and manuscript according to the recommendation of the following references.

[1]. TJ Cole. Setting number of decimal places for reporting risk ratios: rule of four. BMJ. 2015 Apr 27;350:h1845. doi: 10.1136/bmj.h1845.

[2]. European Association of Science Editors. EASE Guidelines for Authors and Translators of Scientific Articles to be Published in English. Acta Inform Med. 2014 Jun;22(3):210-7. doi: 10.5455/aim.2014.22.210-217. Epub 2014 Jun 15. PMID: 25132718; PMCID: PMC4130677.

[3]. TJ Cole. Too many digits: the presentation of numerical data. Arch Dis Child. 2015;100(7):608-9. doi: 10.1136/archdischild-2014-307149. Epub 2015 Apr 15.

***We greatly appreciate your thoughtful comments, which helped to improve the manuscript.***
