# Supplementary material for: Association of menopausal status with body composition and anthropometric indices in Korean women
Source: PLoS One. 2024 May 20;19(5):e0298212. doi: 10.1371/journal.pone.0298212 (PMC11104629; doi:10.1371/journal.pone.0298212)
Supplement: Attachment — Submitted filename: Response to Reviewers-R2-final.docx [file pone.0298212.s002.docx]

Response to Reviewers

To the Reviewers and Editor:

We appreciate the valuable comments that we received regarding our manuscript titled “Association of menopausal status with body composition and anthropometric indices in Korean women”. The manuscript was revised again by an English language editing service (American Journal Experts) to improve the clarity and readability of the language of the paper. We have also revised the manuscript in accordance with the editor’s and reviewers’ comments. Our revisions are listed below.

**Reviewer Comments:**

**Reviewer 2**

Reviewer #2: 1) The body composition and anthropometric indices measurement may be influenced by many confounders, which may influence the results.

**Answer**: We understand the reviewer’s comment that anthropometric indices are influenced by several covariates. Therefore, the covariates were considered in the adjusted models. Specifically, we adjusted for the covariates in models 1 and 2 in this study. For example, Model 2 was adjusted for age, location, occupation, education level, marital status, smoking status, alcohol use, high-level activity, and SBP and DBP levels. The detailed covariates in the models are described in the Statistical analysis subsection and footnotes in Table 2.

2) Diabetes mellitus, dietary habits, familial factors, medications etc. can be the possible confounders which needs addressal.

**Answer**: We agree with the reviewer’s comment. Unfortunately, in our study, there was no information on diseases as confounding variables. Therefore, it is very difficult to use diseases or medications as confounders.

3) What is the clinical implication of the study.

**Answer**: As suggested by the reviewer, we have modified the clinical implications in the last paragraph on page 13. The modified contents are as follows:

“The clinical implication of this study is that after menopause, it is important to consider a decrease in skeletal muscle mass and total body water rather than an increase in obesity or fat mass in any particular region of the body. Although many anthropometric indices and body composition indices were related to menopausal status in our study, these associations disappeared after adjustment for age. Similarly, previous studies have indicated that menopausal status induces a decrease in skeletal muscle mass and total body water intake, irrespective of age [28, 29]. However,”

4) Body composition and total body water may be influenced by the timing of the test due to diurnal and seasonal variations. When the study was performed and how was the inter/intra observer bias in measurement negated?

**Answer**: Body composition and total body water were measured at several hospitals according to the same protocol from 2020 to 2022. Measurements were performed by multifrequency bioelectrical impedance analysis (BIA) devices (Inbody BWA 2.0, Biospace Co. Ltd., Seoul, Korea), and the measurement device automatically calculates body composition values. Recruitment of subjects was carried out over 2 years. Therefore, it was difficult to consider diurnal and seasonal variations in automatic measurements, as in many previous studies. To provide clearer information about body composition measurements, we have modified the contents on body composition measurements in the Anthropometry and body composition measurements subsection. The modified contents are as follows:

“Body composition indices, including total body water, skeletal muscle mass, body fat mass, and body fat percentage, were measured by multifrequency bioelectrical impedance analysis (BIA) devices (Inbody BWA 2.0, Biospace Co. Ltd., Seoul, Korea) while the subjects laid on a bed with clamp-type sensors on both their wrists and ankles according to the measurement protocols and the procedures provided by the manufacturers. After resting for 5 minutes, the device began to measure and automatically calculate body composition.”

5) Ideally the control should be the individual perse. When conducted as a case control study then age, parity matching and including a strict selection criteria are mandatory.

**Answer**: This was a cross-sectional study rather than a case‒control study. We did not mention the prevalence of menopause because it is difficult to consider menopause a disease. Additionally, since the subjects were divided into postmenopausal and premenopausal women, it was difficult to match them according to age. We described the strict selection and exclusion criteria in the second paragraph in the Subjects and Data Source subsection and Figure 1.

***We greatly appreciate your thoughtful comments, which have helped us improve the manuscript.***
